# On the efficiency of chemotactic pursuit - Comparing blind search with temporal and spatial gradient sensing

**DOI:** 10.1038/s41598-019-50514-4

**Published:** 2019-10-01

**Authors:** Claus Metzner

**Affiliations:** Biophysics Group, Department of Physics, Friedrich-Alexander University of Erlangen-Nuremberg, Erlangen, Germany

**Keywords:** Computational biophysics, Computational models

## Abstract

In chemotaxis, cells are modulating their migration patterns in response to concentration gradients of a guiding substance. Immune cells are believed to use such chemotactic sensing for remotely detecting and homing in on pathogens. Considering that immune cells may encounter a multitude of targets with vastly different migration properties, ranging from immobile to highly mobile, it is not clear which strategies of chemotactic pursuit are simultaneously efficient and versatile. We tackle this problem theoretically and define a tunable response function that maps temporal or spatial concentration gradients to migration behavior. The seven free parameters of this response function are optimized numerically with the objective of maximizing search efficiency against a wide spectrum of target cell properties. Finally, we reverse-engineer the best-performing parameter sets to uncover strategies of chemotactic pursuit that are efficient under different biologically realistic boundary conditions. Although strategies based on the temporal or spatial sensing of chemotactic gradients are significantly more efficient than unguided migration, such ‘blind search’ turns out to work surprisingly well, in particular if the immune cells are fast and directionally persistent. The resulting simulated data can be used for the design of chemotaxis experiments and for the development of algorithms that automatically detect and quantify goal oriented behavior in measured immune cell trajectories.

## Introduction

Chemotaxis, the ability of cells to detect and follow concentration gradients of specific chemicals, is ubiquitous in biology (For an introduction to the field, see^1^ and the references therein). It helps sperm cells to find the ovum, directs cell movements during embryo-genesis, but also enables organisms to locate food sources and to avoid hostile environments. In particular, chemotaxis plays a vital role in recruiting motile immune cells to sites of infection or to malignant tumors. This recruitment of immune cells is often based on endogenous chemo-attractants, which are released by other host cells that are already at the location where a pathogen has invaded the body. However, the fact that individual immune cells are able to find and eliminate tumor cells in a Petri dish^2,3^, without any assistance, suggests that immune cells can detect chemical traces emitted by the pathogens themselves. We therefore investigate in this work how efficient a self-propelled agent (such as an immune cell) can hypothetically become in finding and elliminating randomly distributed, mobile targets (such as tumor cells), a problem that is related to the more general topics of pursuit and evasion^4^, to foraging theory^5,6^, to the behavioral ecology of finding resources^7^, and even to robotic control theory^8^.

Chemotaxis does not only play an important role for immune cells and other eukaryotes^9^, but also for prokaryotes, such as bacteria^1,10^. Indeed, the effects of chemotaxis in bacterial systems can be observed even on the macroscopic level. An early example are the traveling bands of chemotactic bacteria, which have been observed already in 1966^11^ and which were theoretically analyzed in the subsequent years^12,13^.

In the first model of chemotaxis, published 1971 by Keller and Segel^14^, both the concentration of the chemoattractant and that of the chemotactic agents is described by continuous distributions, coupled by partial differential equations (PDEs). Subsequently, the Keller-Segel model has been extended^15^, and eventually PDEs became a standard tool for describing chemotactic systems^16^. This framework allowed researchers to investigate a variety of collective effects, such as pursuit-evasion waves in a predator-prey system^17^, or stationary patter formation in a three-species predator-prey model^18^.

Another modeling approach is based on individual chemotactic agents. A simple example is the introduction of chemotactic interactions to Brownian agents, which sometimes leads to analytically treatable models^19^. Compared to PDE-based models, these multi-agent simulations (MAS) are however much more flexible with regard to the properties of the agents and their possible interactions with each other and with the environment (For introductions and critical discussions see^20–23^). MAS can be used to model the chemotactic response of single cells in great detail^24^, but they have also been frequently applied to model the immune response^25–27^, or pattern formation in multi-cellular aggregates and tumor systems^28^. However, the advantages of the PDE and MAS approaches can been combined using hybrid models^29,30^, where the chemotactic agents are described individually, while the chemoattractant is treated as a continuous field.

A kind of hybrid description was also used in a recent theoretical study of Sengupta *et al*.^31^, which investigates the chemotactic pursuit of a single prey agent by a predator. Although this work addresses a research question similar to ours, it is based on different model assumptions. In particular, it assumes not only that the predator is chemically attracted by the prey, but also that the prey is repelled from the predator. Furthermore, the guiding chemicals in^31^ are assumed to have an infinite life time, which prevents the formation of a stable chemical ‘cloud’ around each agent and leads to long-range interactions. Finally, the Sengupta paper is more interested in the dynamics of pursuit and escape, whereas our work is concerned with the efficiency of a repeated search process.

In the literature on search efficiency, an influential paper was published by Viswanathan *et al*. in 1999^32^, in which the authors considered a system where target sites are sparse and can be visited any number of times. They found that search efficiency is maximal for a random walk with an inverse square power-law distribution of flight lengths, corresponding to Levy flight motion. The Levy flight was also found to be advantageous for optimizing the encounter rates between organisms when the searcher is larger or moves faster than the target, and when the target density is low^33^. However, James *et al*.^34^ have demonstrated in 2008 that the simplest random search strategy of all, ballistic motion in a random direction, outperforms a Levy strategy in almost every case. Moreover, Palyulin *et al*.^35^ have argued that the advantages of the Levy walk as a search strategy can easily disappear when the situation is slightly more complex, in particular if there is also a drift term acting on the chemoattractant.

In any case, the Levy walk, due to its scale-free distribution of flight lengths, cannot be applied to cell migration. A much more realistic model for this purpose is the correlated random walk (CRW)^36,37^ with a fixed-scale distribution of step width and a fixed degree of directional persistence. How these two parameters affect the efficiency of blind search has been investigated before. In particular, Bartumeus *et al*.^38^ showed in 2005 that the search efficiency increases monotonically with the degree of directional persistence.

In the present paper, we are mainly interested in the scenario of a single immune cell finding and eliminating several target cells on a two-dimensional plane with periodic boundary conditions. The target cells are modeled as simple agents that move, independently from the immune cell and from each other, with fixed speed and with fixed directional persistence. While migrating, the targets are emitting a chemical substance that acts as a chemo-attractant for the immune cell. This chemo-attractant is assumed to spread quickly within the extracellular medium by linear diffusion. It is also assumed to decay at a constant rate, so that a concentration profile of fixed shape will surround each target cell at any moment.

The immune cell is modeled as a more complex agent with concentration sensors for the chemo-attractant and with the ability to change its migration behavior accordingly. In the simplest case, the immune cell has only a single chemo-attractant sensor and compares the measured local concentrations between subsequent simulation time steps (temporal sensing). In the more powerful case of spatial sensing, the immune cell uses multiple sensors at different body positions to measure the spatial gradient of the chemo-attractant concentration.

In order to modulate the migration properties depending on the sensed concentration gradients, the immune cell uses probabilistic ‘stimulus-response functions’ with tunable parameters. In the case of temporal sensing, the response function controls the momentary probabilities for being in one of two possible modes of migration, characterized by different speeds and degrees of directional persistence. In the case of spatial sensing, the response function determines the probability of the immune cell turning clockwise or counter-clockwise.

The parameters of the response functions are optimized numerically, with the objective to maximize the average number of direct contacts between the immune cell and distinct target cells during a fixed simulation time - a number called the ‘search efficiency’ *Q* (Here, we assume that once a direct contact is established, the respective target cell is immediately removed from the system). In order to obtain an immune cell that is not only efficient in finding specific types of targets but also robust against variable target behavior, the simulated immune cell is confronted with a broad spectrum of target cell speeds *v*_*tar*_ and directional persistences *ε*_*tar*_ during the optimization phase. Once the optimal response parameters are found, we also evaluate the specific performance *Q* = *Q*(*v*_*tar*_, *ε*_*tar*_) of the immune cell as a function of the target cell’s migrational properties.

## Methods

In the following, we describe the different components of the model in detail. An overview of the simulation algorithm is presented in the Supplemental Information [A].

### Cell migration model

We consider a single immune cell (with index *c* = 0) and several target cells (with indices *c* = 1 … *N*_*tar*_) on a two-dimensional simulation area of linear dimension *L*_*sys*_. The migration of the cells is described by the time-dependent position ${\overrightarrow{r}}_{c,n}$ of the respective cell centers, where periodic boundary conditions are applied both in x- and y-direction. Here, *n* is a discrete time index, related to the continuous time by *t*_*n*_ = *n*Δ*t*_*sim*_.

Throughout this work, we use a fixed simulation time interval of

1 $$\Delta {t}_{sim}\,:\,=1\,\min \,.$$

The cell trajectories ${\overrightarrow{r}}_{c,n}$ are modeled as discrete time, correlated random walks. In particular, the update from one position to the next is performed as follows:

2 $${\overrightarrow{r}}_{c,n}={\overrightarrow{r}}_{c,n-1}+{w}_{c,n}\cdot (\begin{array}{l}\cos ({\varphi }_{c,n-1}+{s}_{c,n}|\Delta {\varphi }_{c,n}|)\\ \sin ({\varphi }_{c,n-1}+{s}_{c,n}|\Delta {\varphi }_{c,n}|)\end{array}).$$

In Eq. (2), *w*_*c*,*n*_ is the step width, which is randomly and independently drawn from a Rayleigh distribution with mean value *v*. Note that this corresponds to an average speed of the cell along the contour of the trajectory (which is a sequence of line segments).

The quantity *ϕ*_*c*,*n*−1_ is the planar angle of motion during the last step of cell *c*, that is, $${\varphi }_{c,n-1}=$$$$\arctan (\frac{{y}_{c,n-1}-{y}_{c,n-2}}{{x}_{c,n-1}-{x}_{c,n-2}})$$.

The quantity Δ*ϕ*_*c*,*n*_ is the turning angle between the last and the present step of cell *c*, so that *ϕ*_*c*,*n*_ = *ϕ*_*c*,*n*−1_ + Δ*ϕ*_*c*,*n*_. The turning angles are randomly and independently drawn from a uniform distribution between the limits Δ*ϕ*_*min*_(*ε*) and Δ*ϕ*_*max*_(*ε*). Here, *ε* ∈ [−1, +1] is a persistence parameter, where *ε* = +1 corresponds to fully persistent motion, *ε* = 0 to diffusive motion, and *ε* = −1 to fully anti-persistent motion. Consequently, if *ε* > 0, we define Δ*ϕ*_*min*_(*ε*) = −(1 − *ε*)*π* and Δ*ϕ*_*max*_(*ε*) = +(1 − *ε*)*π*. If *ε* < 0, we define Δ*ϕ*_*min*_(*ε*) = (1 − |*ε*|)*π* and Δ*ϕ*_*max*_(*ε*) = (1 + |*ε*|)*π*. Note that only the magnitude of the turning angle enters in Eq. (2).

The quantity *s*_*c*,*n*_ ∈ {−1, +1} is a sign factor, which controls if the cell moves left (counter-clockwise) of right (clock-wise). It is randomly and independently assigned to one of its two possible values, with a probability *prob*(*R*) = *prob*(*s*_*c*,*n*_ = −1) = *q*_*R*_.

The statistical properties of the random walk generated by Eq. (2) are determined by the three parameters *v*, *ε*, and *q*_*R*_, where *v* controls the speed of the cells, *ε* their directional persistence, and *q*_*R*_ their preference to turn left or right (which is usually balanced, so that *q*_*R*_ = 1/2). In simple cell migration models, these parameters are usually considered as constant over time. However, it has been shown that cell migration is a heterogeneous stochastic process, in which all parameters can change gradually or abruptly, depending on the circumstances of the cell^39,40^. In this work, we assume in particular that the immune cell is able to adapt its speed, persistence and left/right preference in response to local gradients of a chemo-attractant.

### Assumed size and migration parameters of cells

Although this work is not focused on particular types of immune and target cells, we are using size and migration parameters for the simulated cells that are roughly compatible with existing experiments, in particular those involving natural killer cells and K562 leukemia cells^3^.

If not stated otherwise, simulations in this paper assume that both the immune cell and the target cells are rotation-symmetric and have a radius of

3 $${r}_{imm}={r}_{tar}\,:\,=10\,\mu m.$$

Target cells are assumed to be slow and to move diffusively:

4 $${v}_{tar}\,:\,=1\,\frac{\mu m}{min};\,{\varepsilon }_{tar}\,:\,=0.$$

If required, the immune cell is able to move much faster than the targets and, at least for short periods, with perfect directional persistence:

5 $${v}_{imm}\,:\,=0\ldots 6\,\frac{\mu m}{\min };\,{\varepsilon }_{imm}\,:\,=0\ldots 1.$$

### Model for temporal evolution of the chemo-attractant

Our basic proposition is that the target cells emit a substance into the extra-cellular matrix (mainly consisting of water), which is used as a chemo-attractant by the immune cell. For simplicity, we assume that the chemo-attractant is produced at the center point ${\overrightarrow{r}}_{0}$ of each target cell with a constant generation rate *g*. The substance is freely diffusing with diffusion constant *D*, and is spontaneously decaying with a rate *k* (It is important - and also biologically realistic - that this decay rate is non-zero. Otherwise no stationary density profile will develop). This leads to the following partial differential equation for the time-dependent 2D density distribution of the chemo-attractant ${f}_{2D}(\overrightarrow{r},t)$:

6 $$\frac{\partial }{\partial t}\,{f}_{2D}=g\,\delta (\overrightarrow{r}-{\overrightarrow{r}}_{0})+D\,({\nabla }_{x}^{2}+{\nabla }_{y}^{2})\,{f}_{2D}-k\,{f}_{2D},$$

### Typical parameters of diffusion and decay

The diffusion constant of a substance within a liquid medium (here basically water) can be estimated by assuming a spherical shape of the diffusing molecules. Using Stokes formula for the friction force, the resulting Stokes-Einstein relation yields

7 $$D=\frac{{k}_{B}T}{6\pi \eta r},$$

where *T* = 37 °C is the temperature, *η* = 6.91 · 10^−4^ *Pas* is the viscosity of water at this temperature, and *r* is the radius of the diffusing molecule. For a hypothetical molecule with *r* = 3.18 *nm*, one obtains a diffusion constant of

8 $$D\,:\,=100\,\mu {m}^{2}/s=6000\,\mu {m}^{2}/min,$$

which will be used throughout this paper. Note that the same value of *D* was used in an analytical study of the chemo-attractant’s density profile^41^, where the considered molecule was the anaphylatoxin *C*5*a*. Following this reference, we also assume a typical decay constant of

9 $$k\,:\,={10}^{-2}/s=0.6/\min \,.$$

The generation rate *g* is less important in the sense that it does not affect the spatial shape or the temporal evolution of the profile ${f}_{2D}(\overrightarrow{r},t)$.

A dimensional analysis of Eq. (6) reveals that the system has a characteristic diffusion length of

10 $${L}_{dif}=\sqrt{D/k}\approx 100\,\mu m,$$

which can be considered as the approximate spatial extent of the density ‘cloud’ around a stationary emitter. The characteristic time period for developing this density cloud can be estimated as

11 $${T}_{dif}=\frac{{L}_{dif}^{2}}{D}=\frac{1}{k}\approx 100\,s\approx 1.7\,\min \,.$$

### Fast diffusion limit

Based on the above parameters, we can compute a further characteristic quantity that has the dimensions of a velocity:

12 $${v}_{crit}=\frac{{L}_{dif}}{{T}_{dif}}\approx 60\,\mu m/\,\min \,,$$

If the emitter of the density cloud is moving at a speed much smaller than this critical velocity, we can approximately assume that the density cloud is fully developed at any moment in time. In other words, there will be a cloud of fixed (stationary) shape that is ‘carried around’ by the emitter along its trajectory. For our assumed typical target cell speed of *v*_*tar*_ = 1 *μm*/*s*, we are indeed well within this ‘fast diffusion limit’.

### Stationary density profile around single target

**Figure 1:**
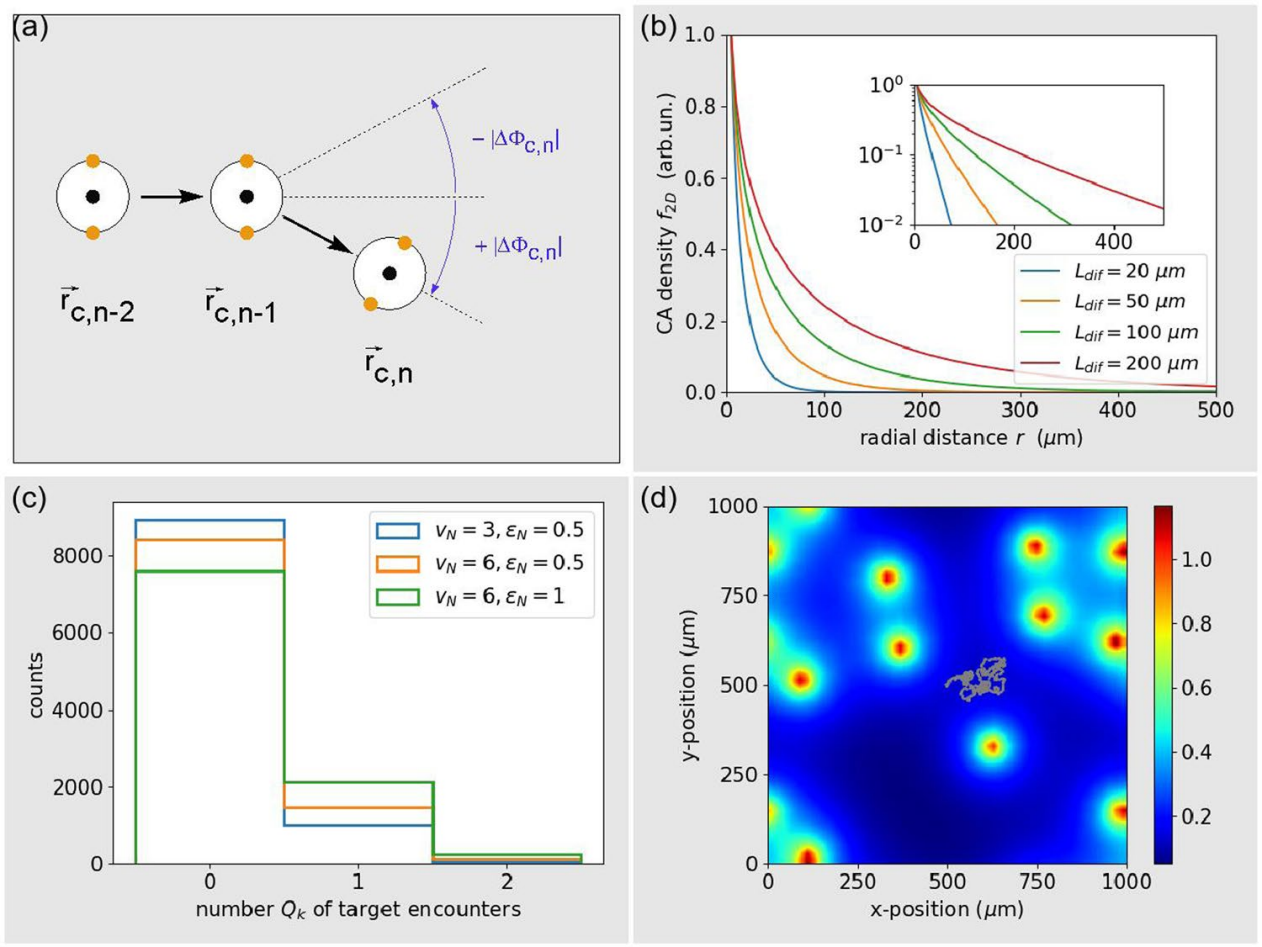
(**a**) Three subsequent positions of the model immune cell (white circles), which is equipped with a central concentration sensor for temporal gradient sensing (black dot) and two lateral concentration sensors for spatial gradient sensing (orange dots). The magnitude of the turning angle |Δ*ϕ*_*c*,*n*_| can be applied with negative of positive sign (blue). (**b**) Stationary radial profile of chemo-attractant density *f*_2*D*_(*r*) around a non-moving emitter, for different diffusion lengths *l*_*dif*_. The semi-logarithmic inset shows that the profile decays almost exponentially for large radial distances *r* → ∞. (**c**) Distribution of the number of targets encountered by the immune cell over 10^5^ simulation runs. The three shown cases correspond to the standard parameters (SP, blue), to standard parameters with the immune cell persistence increased to *ε*_*N*_ = 1 (olive), and to standard parameters with both *ε*_*N*_ = 1 and speed increased to *v*_*N*_ = 6 *μm*/min (red). (**d**) Example configuration of static targets (orange dots), concentration distribution of the guiding substance (color code), and the trajectory of the immune cell (small gray dots) over 500 min. The immune cell is set to standard parameters (*c*_*A*0_ = −5, *c*_*A*1_ = *c*_*R*1_ = 0, *v*_*N*_ = *v*_*A*_ = 3, and *ε*_*N*_ = *ε*_*A*_ = 0.5).

The fast diffusion limit saves us from numerically solving the reaction-diffusion equation Eq. (6). We only need to compute the stationary, rotation-symmetric density profile ${f}_{2D}(r=|\overrightarrow{r}-{\overrightarrow{r}}_{0}|)$ around a non-moving emitter, conveniently located at the origin ${\overrightarrow{r}}_{0}=\overrightarrow{0}$ of the coordinate system. Since the immune cell can never be closer to the emission point ${\overrightarrow{r}}_{0}$ than the radius *r*_*tar*_ of the target cells, we need to solve Eq. (6) only in the region *r* < *r*_*tar*_, where the generation term disappears. In polar coordinates, these simplifications lead to the following ordinary differential equation for the stationary profile,

13 $$[\frac{{\partial }^{2}}{\partial {r}^{2}}\,\,+\frac{1}{r}\,\,\frac{\partial }{\partial r}]\,{f}_{2D}(r)=(\frac{k}{D})\,{f}_{2D}(r),$$

which is solved numerically with a Runge Kutta method. At the border of the target cell, without restriction of generality, we set the density to *f*_2*D*_(*r* = *r*_*tar*_) = 1. The slope $\frac{\partial }{\partial r}{f}_{2D}(r={r}_{tar})$ at this point is iteratively adjusted such that *f*_2*D*_(*r* → ∞) = 0. The resulting radial profile decays rapidly in the direct vicinity of the emitter. Fo*r r* → ∞, the curve approaches an exponential shape (see Fig. 1(b)). Since the density ‘kernels’ *f*_2*D*_(*r*) of different emitters add up linearly, the total distribution of chemo-attractant density from all present target cells can be written as

14 $${F}_{2D}(\overrightarrow{r},t={t}_{n})=\mathop{\sum }\limits_{c=1}^{{N}_{tar}}\,{f}_{2D}(|\overrightarrow{r}-{\overrightarrow{r}}_{cn}|).$$

### Modeling sensors for the chemo-attractant

In the case of *temporal sensing*, we assume that the immune cell can measure, in every time step *n*, the total density ${\rho }_{n}^{C}={F}_{2D}(\overrightarrow{r}={\overrightarrow{r}}_{C},t={t}_{n})$ of chemo-attractant at the center ${\overrightarrow{r}}_{C}={\overrightarrow{r}}_{0,n}$ of its cell body. It then computes the temporal difference

15 $$\Delta {\rho }_{n}^{C}={\rho }_{n}^{C}-{\rho }_{n-1}^{C}.$$

In the case of *spatial sensing*, we assume that the immune cell has two sensors at the left and right border of its cell body, that is, at positions

16 $${\overrightarrow{r}}_{L/R}={\overrightarrow{r}}_{0,n}+{r}_{imm}\cdot (\begin{array}{l}\cos ({\varphi }_{0,n-1}\pm \pi /2)\\ \sin ({\varphi }_{0,n-1}\pm \pi /2)\end{array}),$$

where the corresponding total chemo-attractant densities are *ρ*_*n*_^*L*^ and *ρ*_*n*_^*R*^, respectively. It then computes the spatial difference

17 $$\Delta {\rho }_{n}^{LR}={\rho }_{n}^{R}-{\rho }_{n}^{L}.$$

### Mapping sensor signals to migration behavior

The two sensor signals available to the immune cell are the temporal density difference Δ*ρ*_*n*_^*C*^ and the spatial density difference Δ*ρ*_*n*_^*LR*^. The migration parameters which can be affected by these sensor signals are the speed of the immune cell *v*, its directional persistence *ε*, and its preference to turn left *q*_*L*_.

For simplicity, we assume that the immune cell has two distinct migration modes, called the ‘*normal mode*’ *N*, and the ‘*approach mode*’ *A*. In the normal mode, the speed is *v*_*N*_ and the persistence is *ε*_*N*_. In the approach mode, the speed is *v*_*A*_ and the persistence is *ε*_*A*_. These four parameters can be tuned to optimize search performance.

At any time step *n*, the immune cell can only be in one of these two migration modes. The probability to be in the approach mode is computed as a function of the *temporal* gradient as follows

18 $${q}_{A}=prob(A)=logistic({c}_{A0}+{c}_{A1}\Delta {\rho }_{n}^{C}),$$

where

19 $${logistic}(x)=\frac{1}{1+{e}^{-x}}$$

is the logistic function, and *c*_*A*0_ as well as *c*_*A*1_ are unknown coefficients that also have to be optimized. Note that for *c*_*A*1_ < 0, the mode *A* is favored whenever there is a positive temporal gradient, provided that the magnitude of the bias *c*_*A*0_ is note too large.

In a similar way, the *spatial* gradient determines the probability *q*_*R*_ of the immune cell to turn right:

20 $${q}_{R}=prob(R)=logistic({c}_{R1}\Delta {\rho }_{n}^{LR}),$$

where *c*_*R*1_ is an additional coefficient to be optimized. Note that for *c*_*R*1_ < 0, right turns are favored whenever the chemo-attractant density at the right sensor is larger than that on the left sensor.

### Choice of target cell density and linear system size

The density of target cells in the two-dimensional simulation plane is chosen to be

21 $${\rho }_{tar}\,:\,=1\times {10}^{-5}\,\mu {m}^{-2}.$$

This density leads to a mean distance between nearest neighbors of

22 $${\bar{r}}_{nn}=\frac{1}{2\sqrt{{\rho }_{tar}}}\approx 158\,\mu m,$$

which is slightly larger than the diffusion length *L*_*dif*_ = 100 *μm*.

The linear system size is chosen as

23 $${L}_{sys}=1000\,\mu m,$$

which is considerably larger than *L*_*dif*_ and ${\bar{r}}_{nn}$. The average number of target cells within the simulation area is

24 $${N}_{tar}={\rho }_{tar}{L}_{sys}^{2}=10.$$

Note that if an immune cell is migrating with its maximum speed of 6 *μm*/min and with perfect directional persistence, it would take about 26 min (=26 simulation time steps) to cover the distance between two neighboring target cells. Within 100 min, an immune cell of perfect efficiency might encounter 3 to 4 target cells (ignoring the fact that *r*_*nn*_ is increasing slightly with each encounter and the simultaneous removal of the target).

### Measuring search efficiency

We thus set the time period of a single simulation run to

25 $${T}_{sim}\,:\,=100\,\min \,.$$

After a specific simulation run *k*, the number of remaining target cells *N*_*tar*,*k*_^*rem*^ is counted. We then quantify the efficiency of the immune cell by the number of eliminated target cells:

26 $${Q}_{k}={N}_{tar}-{N}_{tar,k}^{rem},$$

a quantity that can fluctuate considerable between each run. To overcome these fluctuations, the simulation is repeated

27 $${N}_{runs}\,:\,={10}^{4}$$

times for each set of system parameters, using in each run a random initial configuration of the single immune cell and of the *N*_*tar*_ = 10 target cells.

Finally, the *search efficiency* of the immune cell is defined as the average

28 $$Q=\frac{1}{{N}_{runs}}\mathop{\sum }\limits_{k=1}^{{N}_{runs}}\,{Q}_{k}.$$

### Effect of periodic boundary conditions

The periodic boundary conditions used in the simulations will not significantly affect the results, as long as the linear system size (*L*_*sys*_ = 1000 *μm*) is large compared to the other characteristic length scales of the problem, such as the mean distance between nearest neighbors $\bar{r}$_*NN*_ ≈ 158 *μm*, or the directional correlation length *L*_*dir*_ of the cells (corresponding to the ‘persistence length’ in polymer science). While *L*_*sys*_ ≪ *L*_*dir*_ for modest values of the persistence parameter *ε*, the directional correlation length *L*_*dir*_ grows to infinity as *ε* approaches one.

In (or close to) this extreme case of ballistic motion, the periodic boundary conditions can lead to unrealistic results. For example, a cell traveling ballistically along a rational migration direction *ϕ* will actually perform a periodic orbit, and thus visit over infinite time only a finite amount of space. However, since our simulation time is *T*_*sim*_ = 100 *min*, even a cell of maximum speed *v* = 6 *μ*/*min* will only cover 60 percent of the linear system size *L*_*sys*_ = 1000 *μm*.

### Optimization of response parameters

In general, the search efficiency in our model depends on up to *n* = 7 unknown parameters *π*_*i*_:

29 $$Q=Q(\overrightarrow{\pi })=Q({v}_{N},{\varepsilon }_{N},{v}_{A},{\varepsilon }_{A},{c}_{A0},{c}_{A1},{c}_{R1}).$$

Finding the search strategy with the best search efficiency amounts to finding the parameter combination $\overrightarrow{\pi }$ that maximizes *Q*:

30 $${\overrightarrow{\pi }}_{opt}={argmax}\{Q(\overrightarrow{\pi })\}$$

We perform this quite high-dimensional numerical optimization using a grid-based variant of the ‘Cyclic Coordinate Descent’ method (CCD, see^42^). In each loop of this iterative method, the *n* parameters/coordinates *π*_*i*_ _∈_ _[1_…_*n*]_ are optimized one after the other in a cyclic way, greedily keeping the remaining *n* − 1 coordinates at their presently best-performing values. In our variant of the method, an individual parameter *π*_*k*_ is optimized by evaluating *Q* = *Q*(*π*_*k*_, {*π*_*i*≠*k*_}) for all discrete values of *π*_*k*_ on a regular grid within predefined minimum and maximum values, that is *π*_*k*_ ∈ [*π*_*k*,*min*_,*π*_*k*,*min*_ + Δ*π*_*k*_, …, *π*_*k*,*max*_] The method stops when the same set of *n* optimal parameters is found in two subsequent iteration loops.

### List of standard parameters

In Table 1, we provide a list of all relevant system parameters, here called the Standard Parameters (SP). The first 12 parameters of the list are fixed for all simulations. During the optimization phase, a different random value of *v*_*tar*_ and *ε*_*tar*_ is drawn for each target cell, from uniform distributions in their respective ranges. During the evaluation phase, all *N*_*tar*_ target cells are set to the same values of *v*_*tar*_ and *ε*_*tar*_, and these two parameters are then scanned through their ranges in subsequent simulation runs. The last 7 parameters of the list (*v*_*N*_, *ε*_*N*_, *v*_*A*_, *ε*_*A*_, *c*_*A*0_, *c*_*A*1_, *c*_*R*1_) are free to be optimized within the given ranges.

**Table 1:** Table of standard, fixed simulation parameters, and the free parameters that can be optimized (last seven rows).

Symbol	Value	Unit	Description
*L*_*sys*_	1000	*μ*m	Linear system size
Δ*t*_*sim*_	1	min	Simulation time step
*T*_*sim*_	100	min	Total simulation time per run
*N*_*runs*_	10000	—	Number of runs per parameter set
*D*	6000	*μ*m^2^/min	Diffusion constant of chemo-attractant
*k*	0.6	1/min	Decay rate of chemo-attractant (CA)
*N*_*tar*_	10	—	Initial number of target cells
*r*_*tar*_	10	*μ*m	Radius of target cells
*v*_*tar*_	[0, 6]	*μ*m/min	Speed of target cells, uniformly distributed
*ε*_*tar*_	[0, 1]	—	Persistence of target cells, uniformly distributed
*N*_*imm*_	1	—	Number of immune cells
*r*_*imm*_	10	*μ*m	Radius of immune cell
*v*_*N*_	[0, 6]	*μ*m/min	Speed of immune cell in normal mode
*v*_*A*_	[0, 6]	*μ*m/min	Speed of immune cell in approach mode
*ε*_*N*_	[0, 1]	—	Persistence of immune cell in normal mode
*ε*_*A*_	[0, 1]	—	Persistence of immune cell in approach mode
*c*_*A*0_	[−5, 5]	—	Bias of immune cell for approach mode
*c*_*A*1_	[−500, 500]	—	Sensitivity of immune cell for temporal CA differences
*c*_*R*1_	[−500, 500]	—	Sensitivity of immune cell for spatial CA differences

Throughout this paper, we implicitly assume that all fixed parameters are set according to this table.

### Covered area

In order to compute the search area that an immune cell is exploring over time, we partition the 2D simulation space into quadratic patches of linear size *δL* = 10 *μm*. The covered area *A*(*t*) of an immune cell is then defined as *A*(*t*) = *n*_*vis*_(*t*)*δL*^2^, where *n*_*vis*_(*t*) is the total number of patches that the immune cell has visited (at least once) in the time period [0, *t*].

### Mean squared displacement

An important property of random walks is the mean squared displacement $\overline{{R}^{2}}(\Delta t)$ as a function of lag-time Δ*t*. It is defined as

31 $$\bar{{R}^{2}}(\Delta t)={\langle {(\overrightarrow{r}(t+\Delta t)-\overrightarrow{r}(t))}^{2}\rangle }_{t},$$

where the average is over all time steps *t*, and $\overrightarrow{r}(t)$ is the position of a cell at time *t* = *n*Δ*t*_*sim*_. Note that for the purpose of computing $\overline{{R}^{2}}(\Delta t)$ of a cell, we re-calculate its trajectory from the individual steps, using Eq. (2), but without applying periodic boundary conditions.

### Distribution of nearest target distances

If an immune cell is surrounded by a density *ρ*_*tar*_ of randomly located targets, and if there is no correlation between the immune and target cells, the probability density *p*(*d*_*NN*_) of finding the nearest target at a distance *d*_*NN*_ is given by the Rayleigh distribution *p*(*d*_*NN*_) = 2*πρ*_*tar*_ *d*_*NN*_ exp(−*πρ*_*tar*_*d*_*NN*_^2^). By contrast, if the positions of the immune and target cells are somehow correlated, the distribution *p*(*d*_*NN*_) may change. In order to obtain *p*(*d*_*NN*_) numerically, we determine the momentary nearest target (assuming periodic boundary conditions) for each immune cell in each simulation time step, store the distances *d*_*NN*_ to these nearest targets in a list, and finally compute a histogram of the stored values.

### Third party rights

All material used in the paper are the intellectual property of the authors.

## Results

### Concentration profile of chemo-attractant

In the fast diffusion limit, the global concentration distribution of chemo-attractant is a linear superposition of ‘kernels’, centered around the target cells. These kernels are the temporally stationary, rotationally symmetric solutions *f*_2*D*_(*r*) of Eq. (13). We have numerically computed the kernel for different diffusion lengths *L*_*dif*_ (See Fig. 1(b), in which the the green line corresponds to the case of standard parameters). As expected, the concentration profile decays almost exponentially for large radial distances *r* → ∞.

### Blind search (BLS)

We start with an immune cell that completely lacks the ability to sense concentration gradients (*c*_*A*1_ = *c*_*R*1_ = 0), and which is therefore performing a ‘blind’ search process (BLS). At the same time, we assume an extreme bias for the normal migration mode (*c*_*A*0_ = −5), which reduces the probability of the immune cell being spontaneously in the approach mode to an almost negligible value of *q*_*A*_ ≈ 0.007 (The migration parameters of the approach mode are set to medium values *v*_*A*_ = 3 and *ε*_*A*_ = 0.5). The migration of such an immune cell can therefore be described as a homogeneous, correlated random walk with a fixed speed *v*_*N*_ and a fixed degree of directional persistence *ε*_*N*_.

The *N*_*tar*_ = 10 target cells, which are assigned random positions and migration directions before each simulation run, are assumed to form a widely mixed ensemble with respect to their migration parameters. For this purpose, at the beginning of each simulation run, we draw the speed and persistence parameters of each target cell independently from uniform distributions in the ranges *v*_*tar*_ ∈ [0, 6] and *ε*_*tar*_ ∈ [0, 1], respectively.

We first set the migration parameters of the immune cell to medium values *v*_*N*_ = 3 and *ε*_*N*_ = 0.5. In this case, the trajectory of the immune cell is not able to explore a significant part of the simulation area, even when the available time span is increased from the standard setting *T*_*sim*_ = 100 min to *T*_*sim*_ = 500 min (See Fig. 1(d)). Repeating the simulation *N*_*run*_ = 10^4^ times, each spanning an evaluation period of *T*_*sim*_ = 100, we find that the number *Q*_*k*_ of encounters between the immune cell and target cells is fluctuating from one run *k* to the next. The distribution *p*(*Q*_*k*_) has an approximately exponential shape: In most simulation runs, the immune cell does not find any target, rarely one target, and almost never two targets. The average number of encounters with target cells, defined above as the search efficiency, is *Q* = 0.110 in this case. If we let the immune cell migrate faster, using the parameters *v*_*N*_ = 6 and *ε*_*N*_ = 0.5, the search efficiency increases to *Q* = 0.173. Additionally making the immune cell more directionally persistent, using the parameters *v*_*N*_ = 6 and *ε*_*N*_ = 1, results in a further increase of the search efficiency to *Q* = 0.271. This demonstrates that even a blind, homogeneous search process can be optimized via the migration parameters *v*_*N*_ and *ε*_*N*_.

**Figure 2:**
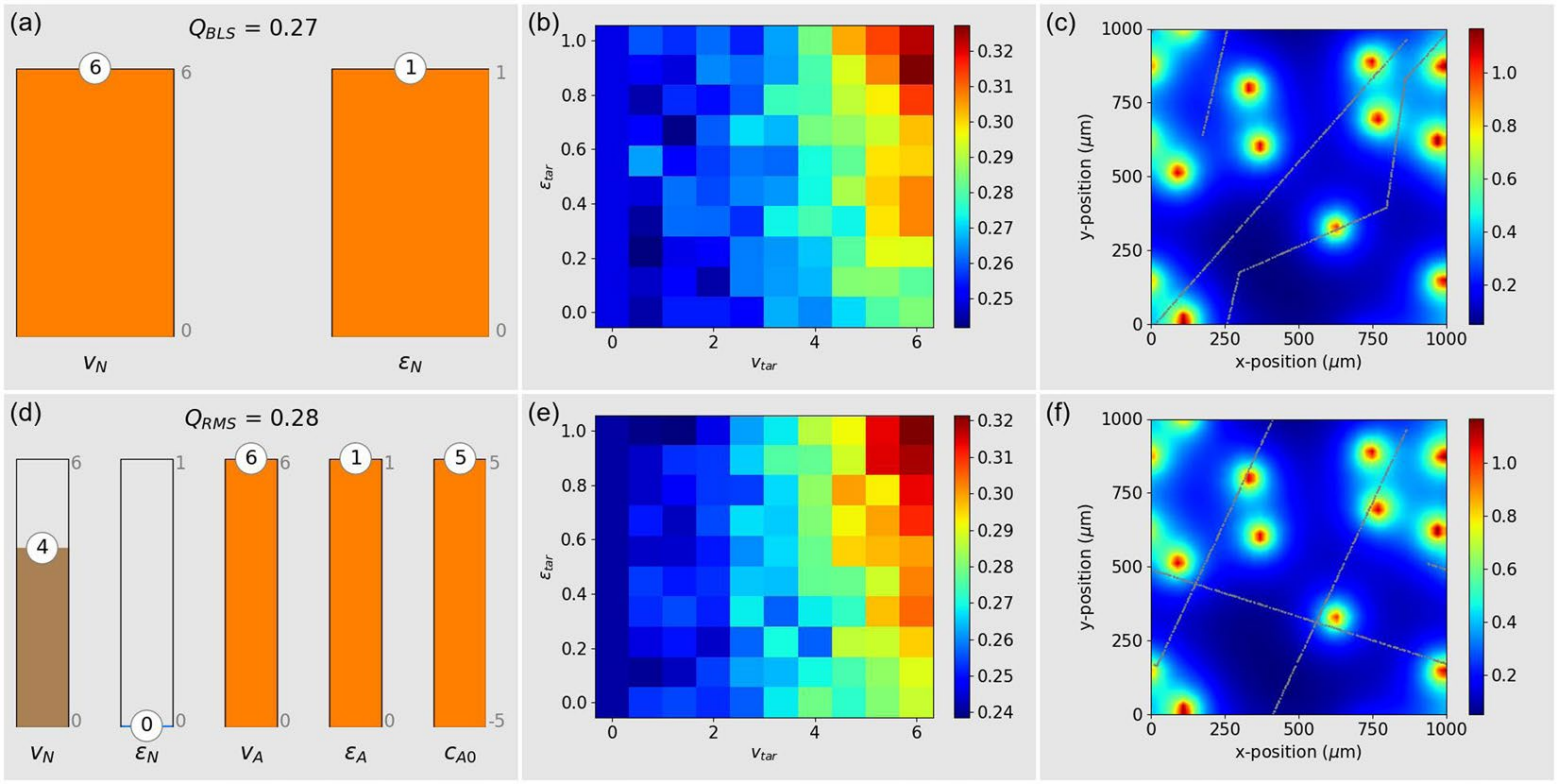
Blind search BLS (**a**–**c**) and Random Mode Switching (**d**–**f**). Left column (**a**,**d)**: Optimum parameters for an immune cell that faces a mixed ensemble of targets with random speeds and persistences. Middle column (**b**,**e**): Search efficiency of the optimized immune cell when facing a pure ensemble of targets with fixed speed *v*_*tar*_ and fixed persistence *ε*_*tar*_. Right column (**c**,**f**): Example configuration of static targets (orange dots), concentration distribution of the guiding substance (color code), and trajectory of the immune cell (small gray dots) over an extended period of 500 min.

We therefore use CCD optimization to find the perfect migration parameters for the immune cell, again using the mixed ensemble of target cells throughout the optimization phase. It turns out that a *blind, homogeneous search within a mixed ensemble of targets has the best efficiency Q when it is performed with maximum possible speed (in our case v*_*N*_ = *6*) *and with perfect directional persistence ε*_*N*_ = 1 (Fig. 2(a)).

The resulting optimal efficiency *Q*_*BLS*_ = 0.27 can be seen as the overall performance of the immune cell, averaged over many possible types of target cells. In practice, it will also be of interest how the immune cell is performing against targets with specific, fixed migration parameters. To investigate this ‘versatility’ of the immune cell, we have computed the search efficiency *Q* = *Q*(*v*_*tar*_, *ε*_*tar*_) of the optimized immune cell (that is, using *v*_*N*_ = 6 and *ε*_*N*_ = 1), as a function of the speed and persistence of the *target* cell (Fig. 2(b)). Here we find that the resulting search efficiency can vary between *Q*_*min*_ ≈ 0.25 and *Q*_*max*_ ≈ 0.32, depending on these two parameters. In particular, *blind, homogeneous search works best when the targets are themselves fast and directionally persistent*. Yet, if the targets exceed the immune cell with respect to the migration parameters, it is more appropriate to say that the targets are finding the immune cell than vice versa.

It is instructive to inspect the trajectory of the immune cell (Small gray dots in Fig. 2(c)), in relation to the targets, over an extended time period. For this purpose, we set the speed of the targets to zero, so that they remain stationary throughout the entire simulation. Since the persistence of the optimized immune cell is *ε*_*N*_ = 1 in the normal mode, the trajectory is straight for most of the time (Note the effect of periodic boundary conditions). However, with a tiny probability of *q*_*A*_ ≈ 0.007, the immune cell also adopts the ‘approach mode’, where the migration parameters are *v*_*A*_ = 3 and *ε*_*A*_ = 0.5, and these rare events lead to an abrupt change of direction. It is remarkable that there occur several ‘near misses’ between the immune cell and one of the targets. Yet, without any sensing abilities, the immune cell most of the time cannot seize these opportunities.

### Random mode switching (RMS)

We continue to consider blind search, characterized by the absence of sensitivity for concentration gradients (*c*_*A*1_ = *c*_*R*1_ = 0). But this time we allow the immune cell to switch between its two migration modes randomly and spontaneously, a situation that creates a heterogeneous correlated random walk. For this purpose, we now declare not only the parameters *v*_*N*_ and *ε*_*N*_, but also *c*_*A*0_, *v*_*A*_ and *ε*_*A*_ as free, optimizable parameters.

Although the system is now considerably more flexible than in the case of homogeneous blind search, CCD optimization shows that this flexibility brings no significant improvement of the the search efficiency (Fig. 2(d)), as *Q*_*RMS*_ = 0.28 ≈ 0.27 = *Q*_*BLS*_. Indeed, the optimal efficiency is found for a bias *c*_*A*0_ = 5, which keeps the immune cell in the approach mode virtually all the time, thus leaving the values *v*_*N*_ = 4 and *ε*_*N*_ = 0 irrelevant. Within the approach mode, the optimized immune cell is as fast (*v*_*A*_ = 6) and persistent (*ε*_*A*_ = 1) as possible, just like in the above homogeneous BLS case. This demonstrates that *in blind search, purely spontaneous mode switching performs worse than a homogeneous random walk at maximum speed and perfect directional persistence*. Since the optimal RMS strategy is - except for a name change of the dominating migration mode - identical to the BLS strategy, we also find the same results for *Q* = *Q*(*v*_*tar*_, *ε*_*tar*_) (Fig. 2(e)). The sample trajectory of the immune cell also resembles that of the BLS strategy (Fig. 2(f)).

### Temporal gradient sensing (TGS)

Next, we investigate how the killing efficiency can be enhanced when the immune cell is able to measure temporal gradients of the chemo-attractant and to switch between the normal mode *N* and the approach mode *A* accordingly. In order to make this adaptive mechanism work, there are six parameters to be optimized: The speeds (*v*_*N*_, *v*_*A*_) and persistence values (*ε*_*N*_, *ε*_*A*_) in the two migration modes, as well as the bias of the approach mode (*c*_*A*0_) and the sensitivity for temporal chemo-attractant gradients (*c*_*A*1_). Without restriction of generality, the latter quantity is assumed to be non-negative, *c*_*A*1_ ≥ 0, because a positive temporal gradient of the chemo-attractant Δ*ρ*_*n*_^*C*^ means that the immune cell is approaching a target cell, and this should increase the probability of the approach mode *A*, whatever this means for the speed and persistence of the immune cell.

**Figure 3:**
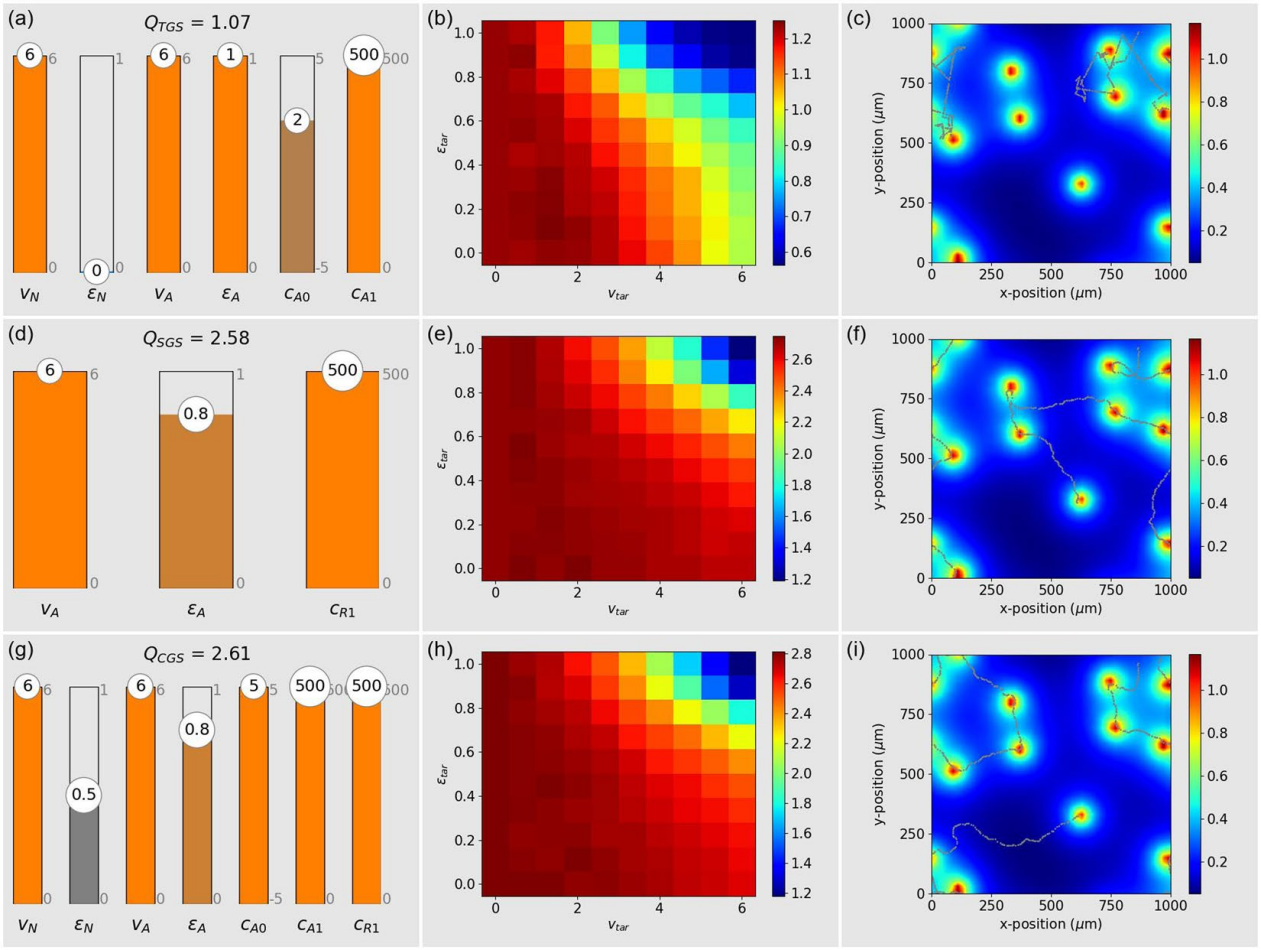
Temporal Gradient Search TGS (**a**–**c**), Spatial Gradient Search SGS (**d**–**f**), and Combined Gradient Search CGS (**g**–**i**). Left column (**a**,**d**): Optimum parameters for an immune cell that faces a mixed ensemble of targets with random speeds and persistences. Middle column (**b**,**e**): Search efficiency of the optimized immune cell when facing a pure ensemble of targets with fixed speed *v*_*tar*_ and fixed persistence *ε*_*tar*_. Right column (**c**,**f**): Example configuration of static targets (orange dots), concentration distribution of the guiding substance (color code), and trajectory of the immune cell (small gray dots) over an extended period of 500 min.

CCD optimization shows (Fig. 3(a)) that the optimum bias for the approach mode is *c*_*A*0_ = 2, which corresponds to a probability *q*_*A*_ ≈ 0.88 of the immune cell being in the approach mode if detecting no or only a very weak temporal gradient. When however a significant gradient is present, the large sensitivity parameter *c*_*A*1_ = 500 causes an almost deterministic mode switching behavior: *In positive gradients, the optimal TGS cell is adopting the approach mode, which is maximally fast (v*_*A*_ = *6) and persistent (ε*_*A*_ = 1*). In negative gradients, it is adopting the normal mode, which is also fast (v*_*N*_ = *6), but directionally non-persistent (ε*_*N*_ = 0*)*. The resulting search efficiency of the optimized TGS strategy against target cells with mixed migration properties is *Q*_*TGS*_ = 1.07, which surpasses the blind strategies by a factor of *Q*_*TGS*_/*Q*_*BLS*_ ≈ 4.

Confronted with target cells of fixed migration properties (Fig. 3(b)), the performance of the optimized TGS strategy is degrading relatively quickly when the targets are fast and directionally persistent.

The sample trajectory of the immune cell (Fig. 3(c)) demonstrates the alternating phases of zero persistence (*ε*_*N*_ = 0, ‘zigzag’-like motion) and perfect persistence (*ε*_*A*_ = 1, straight motion). In contrast to the blind search strategies, the immune cell is now able to perfectly home in on a target, once it came close to it.

### Spatial gradient sensing (SGS)

We now consider an immune cell that is virtually always in the approach mode (enforced by *c*_*A*0_ = 500), but has the ability to turn left (clock-wise) or right in response to the spatial gradient of the chemo-attractant. The relevant response coefficient for this mechanism is the sensitivity *c*_*R*1_. Yet, how well the immune cell can follow a spatial gradient also depends on the migration parameters *v*_*A*_ and *ε*_*A*_, because they determine how quickly the cell can adjust its direction as it follows a spatial gradient.

CCD optimization shows (Fig. 3(d)) that *the optimized SGS immune cell turns into the direction of larger chemo-attractant concentration with maximum sensitivity (c*_*R*1_ = *500). It migrates with maximal speed (v*_*A*_ = *6), but with a specific degree of persistence that is smaller than one* (*ε*_*A*_ = 0.8). The resulting search efficiency of the optimized SGS strategy against target cells with mixed migration properties is *Q*_*SGS*_ = 2.58, which surpasses the TGS strategy by a factor of *Q*_*SGS*_/*Q*_*TGS*_ ≈ 2.4, and blind strategies by a factor of *Q*_*SGS*_/*Q*_*BLS*_ ≈ 9.6.

Confronted with target cells of fixed migration properties (Fig. 3(e)), the optimized SGS cell has a relatively constant performance for targets with small to medium speeds and persistences. In the extreme case of targets with *v*_*tar*_ ≈ 6 and *ε*_*tar*_ ≈ 1, the performance declines, but even then it is still about as good as the optimal TGS performance.

The sample trajectory (Fig. 3(f)) shows that the optimized SGS immune cell is wasting almost no time between subsequent target attacks. It moves from one target to the next in an efficient way, resembling the optimal solutions of a traveling salesman problem.

### Combined spatial and temporal gradient sensing (CGS)

We also consider an immune cell that can, both, switch between two migration modes in response to the temporal chemo-attractant gradient, and at the same time turn left and right in response to the spatial gradient. Since these two mechanisms have different requirements with respect to the migration parameters (For example, temporal sensing requires *ε*_*N*_ = 0, but spatial sensing works best with *ε*_*N*_ = 0.8), it is not clear whether a combination of the two abilities is advantageous or reduces the killing efficiency.

CCD optimization of combined gradient sensing involves the complete set of eight free parameters (Fig. 3(g)). The resulting bias *c*_*A*0_ = 5 means that the optimized CGS cell is adopting the approach mode practically all the time. In this mode, it just performs spatial gradient sensing, since all the parameters that are relevant to SGS are actually unchanged (*c*_*R*1_ = 500, *v*_*A*_ = 6, and *ε*_*A*_ = 0.8). However, the optimized CGS cell is also highly sensitive to temporal gradients (*c*_*A*1_ = 500). Therefore, in the presence of a sufficiently negative temporal gradient, it will switch to the normal mode, which is fast (*v*_*N*_ = 6) but only medium persistent (*ε*_*N*_ = 0.5). This means that *Combined gradient sensing is basically like spatial gradient sensing, but with the additional feature of a less persistent migration in strongly negative temporal gradients*. The resulting search efficiency of the optimized CGS strategy against target cells with mixed migration properties is *Q*_*CGS*_ = 2.61, which is only slightly better than the SGS strategy. The versatility of combined gradient sensing resembles that of purely spatial gradient sensing (Fig. 3(h)). Also the sample trajectory (Fig. 3(i)) has basically the same characteristics as in the SGS strategy.

### Indirect effects of target-directed search

We have shown above that a target-directed migration of the immune cells, as realized in the TGS, SGS and CGS strategies, significantly improves the search efficiency *Q* compared to random migration. However, it is not clear how the target-directed behavior affects other statistical properties of the immune cell trajectories. We therefore investigate in the following the effect of TGS and SGS on three selected properties that are relevant in the context of search.

**Figure 4:**
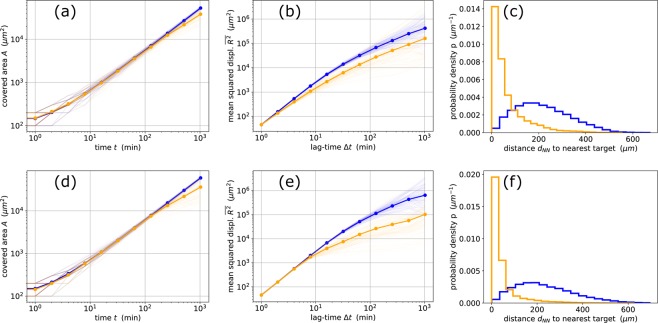
Indirect effects of goal-directed search on the immune cell’s covered area A(t) versus time (first column), on its mean squared displacement $\overline{{R}^{2}}(\varDelta t)$ versus lag-time (second column), and on the probability density *p*(*d*_*NN*_) of the distance to the nearest target (third column). The top row (**a**–**c**) corresponds to temporal gradient search (TGS), the bottom row (**d**–**f**) to spatial gradient search (SGS). Orange lines were obtained with the optimized immune cell parameters, whereas blue lines were obtained with sensitivity parameters (*c*_*A*1_ for TGS, and *c*_*R*1_ for SGS) set to zero, effectively creating a blind search. In the double-logarithmic plots of the first two columns, the fine lines correspond to individual immune cells (10 per run) and simulation runs (10), whereas the thick lines are logarithmic averages. The search strategies TGS and SGS have little effect on the immune cell’s covered area ((**a**,**d**)), but lead to significantly reduced mean squared displacements at longer lag-times ((**b**,**e**)). The most drastic effect of TGS and SGS is seen in the distribution *p*(*d*_*NN*_), which is Rayleigh-like in blind search (blue histograms in (**c**,**f**)), but exponential-like in goal-directed search (orange histograms in (**c**,**f**)). These effects are caused by the attraction of the immune cells towards the targets, which in turn leads to a partial localization of the immune cell trajectory in the vicinity of targets.

First, we consider how the total search area *A*(*t*) explored by an immune cell is growing over time, both in target-directed search and in a blind reference search with comparable parameters. Note that in a blind search, if targets are distributed randomly (that is, according to a spatial Poisson distribution with a fixed average density) and are not moving, the only way to improve the search efficiency *Q* is to increase *A*(*t*) as fast as possible. In the optimal case (corresponding to hypothetical immune cells that migrate along a straight line and with constant velocity through an infinite territory, thereby avoiding to ever return to the same spot again), the search area *A*(*t*) would increase linearly with time. Indeed, we find an almost linear increase of *A*(*t*) for immune cells that act according to the optimized TGS or SGS parameters, but with the sensitivity for the chemotactic gradients set to zero (blue lines in Fig. 4(a,d)). When the sensitivity is switched back on, the *A*(*t*) curves hardly change, except for very long times *t*, where the target-directed immune cells cover a slightly *smaller* search area than in the blind reference case (orange lines in Fig. 4(a,d)). The reason for the reduced *A*(*t*) is that chemotactically sensitive immune cells spend a longer time in the vicinity of particular targets. In order to home in on these targets, they repeatedly change speed, persistence, or direction, which necessarily reduces their ability to explore new territory.

Next, we consider the immune cell’s mean squared displacement $\overline{{R}^{2}}(\Delta t)$ (abbreviated as MSD) as a function of lag-time, which is an important statistical property in the theory of random walks (Note that for the purpose of computing the MSD, the immune cell trajectories were re-constructed without periodic boundary conditions). In the case of the ‘blinded’ reference cells (blue lines in Fig. 4(b,e)), we find the typical MSD signature of a directionally persistent random walk with a fixed velocity auto-correlation time: A gradual cross-over from ballistic behavior ($\overline{{R}^{2}}\propto \Delta {t}^{2}$) at small lag-times to diffusive behavior ($\overline{{R}^{2}}\propto \Delta t$) at large lag-times. In the case of target-directed search (orange lines in Fig. 4(b,e)), $\overline{{R}^{2}}(\Delta t)$ is reduced, because the interaction with the target cells effectively decreases the velocity auto-correlation time, so that the non-ballistic regime is starting earlier. In the case of SGS (orange lines in Fig. 4(e)), the MSD at large lag-time is even slightly sub-diffusive, reflecting the fact that the immune cells get ‘bound’ to their targets for a certain time period.

Finally, we consider the distribution *p*(*d*_*NN*_) of distances between an immune cell and its nearest target. In an infinite system where immune and target cells are distributed randomly with fixed average densities, without any statistical correlations between the cell positions, *p*(*d*_*NN*_) would be a Rayleigh distribution (see Method section), with a peak at some non-zero distance *d*_*NN*_ that depends on the target density as *d*_*NN*_ ∝ *ρ*_*tar*_^−1/2^. This is basically what we find for the ‘blinded’ reference cells (blue lines in Fig. 4(c,f)), although the histograms are not exactly Rayleigh distributions, due to the effect of periodic boundary conditions. By contrast, target-directed search creates exponential-like distributions *p*(*d*_*NN*_) (orange lines in Fig. 4(c,f)), where the most probable distance is approximately zero. This qualitative change of the distribution type demonstrates very drastically the ‘localization’ effect of the chemotactically sensitive immune cells around their momentary targets.

## Discussion and Summary

The present work was motivated by experiments on chemotactic ‘pursuit’ in a Petri dish. These experiments studied the interaction of natural killer (NK) cells^43^ with 293T embryonic kidney cells^2^ or K562 leukemic cells^3^ as targets. These *in-vitro* experiments demonstrated that single immune cells are able to find their targets on their own account. However, it remained unclear if the observed attacks were merely chance encounters, or actually guided by chemotactic mechanisms. Moreover, we do not know which search efficiencies can be expected from a ‘blind’ search, and from different ‘guided’ search strategies based on chemotaxis. Finally, identifying distinct, efficient and robust search strategies by numerical optimization of a simulated immune cell will also reveal characteristic patterns of search behavior, that might then be used as characteristic ‘fingerprints’ of goal-directed search in future automatic detection algorithms.

We have compared five distinct strategies of search, namely blind search with fixed speed and directional persistence of the immune cells (BLS), blind search with random switching between two distinct migration modes (RMS), guided search based on temporal gradients of the chemo-attractant (TGS), guided search based on spatial gradients of the chemo-attractant (SGS), and a combination of temporal and spatial sensing (CGS). Throughout our study, we have kept the system geometry (two dimensions, as on a Petri dish) and all parameters (density of the target cells, properties of the chemo-attractant, sizes and migration properties of the cells, sensing abilities of the immune cell) close to experimentally realistic values.

In the case of blind search (BLS), not surprisingly, the search efficiency of the immune is almost an order of magnitude lower than with the best guided search mechanism. Nevertheless, since many pathogens will not emit any chemical substance that the immune cell can detect and use as a guide to its target, blind search may often be the only option. It is therefore fortunate that blind search can be easily optimized by making the immune cell as fast and directionally persistent as possible. This can be understood most easily assuming immobile target cells that are located at random positions within the plane. As the search time *t* is going on, the blindly migrating immune cell is exploring more and more regions of the Petri dish, and we can mentally mark all spatial pixels that have been visited at least once by the immune cell. The total area of all marked pixels, *A*(*t*), here called the ‘covered area’, is growing monotonously with time, and all target cells that happen to be located within the covered area can be considered as found by the immune cell. Their expected number is $\bar{N}$_*found*_(*t*) = *ρ*_*tar*_ *A*(*t*), where *ρ*_*tar*_ is the areal density of target cells. If the immune cell is migrating with low directional persistence, it will re-visit many pixels more often than once, which is counter-productive with target cells that never move. In this case, the covered area will grow sub-linearly with time. By contrast, *A*(*t*) ∝ *t* for an immune cell that is migrating with perfect directional persistence and constant speed, that is, uniformly along a straight line (See 1.11 for the effect of periodic boundary conditions). It is therefore clear that high directional persistence is an important way to improve the blind search efficiency *Q* of immune cells. At the same time, speed is another key factor for efficient search: For an immune cell in uniform motion, the expected number of found target cells at the end of the search period, $\bar{N}$_*found*_(*t* = *T*_*sim*_) ∝ *v*_*imm*_, will be directly proportional to its speed.

However, it is well-known that actual cells - and not only immune cells - are showing gradual or abrupt changes of their speed and persistence^39,40^, so that their migration has to be described by a temporally heterogeneous stochastic process. The result of such parameter fluctuations are often ‘anomalous’ properties of the cell’s random walk, such as a mean squared displacement that increases with lag-time approximately as a power-law. It is not clear whether temporally heterogeneous cell migration is just a side effect of other causes (such as differences in the local micro-environment of the migrating cell or internal changes connected with the cell cycle), or if it actually serves a purpose. Theoretically, the heterogeneity may help to increase the blind search efficiency of an immune cell, particularly when the targets are mobile. We have therefore investigated how the search efficiency is affected when the immune cell performs random switches between two different migration modes (the RMS strategy). Yet, as suggested by the theoretical argument above, the RMS strategy did not perform significantly better than blind search with fixed migration parameters.

Next, we have investigated guided search strategies that are based on the sensing of chemotactic gradients. In the case of temporal gradient sensing (TGS), we found that the optimized immune cell is switching between two distinct migration modes: In positive gradients, it is adopting the approach mode, which is maximally fast (*v*_*A*_ = 6) and persistent (*ε*_*A*_ = 1). By this way, the cell is climbing up the gradient consistently, which usually corresponds to approaching one of the targets. In negative gradients, it is adopting the normal mode, which is also fast (*v*_*N*_ = 6), but directionally non-persistent (*ε*_*N*_ = 0). In this mode, the cell is exploring new migration directions, until it finds one with a positive gradient. Note that the optimal TGS strategy found here by numerical parameter optimization strongly resembles the chemotaxis behavior of *Escherichia Coli*^44^, with its gradient-dependent switching between swimming and tumbling modes of migration. Compared to blind search, TGS is more effective. On the other hand, the gained factor of four in search efficiency is not really large.

In the case of spatial gradient sensing (SGS), we found that the optimized immune cell turns into the direction of larger chemo-attractant concentration with maximum sensitivity (*c*_*R*1_ = 500). It migrates with maximal speed (*v*_*A*_ = 6), but with a specific degree of persistence that is smaller than one (*ε*_*A*_ = 0.8). Presumably, this specific degree of persistence represents an optimal compromise between the need to maximize the visited area, and the need to perform clockwise and counter-clockwise turns with the right curvature. Compared to blind search, SGS is almost an order of magnitude more efficient. A combination of temporal and spatial sensing (CGS) turned out to bring no significant advantages compared to pure spatial sensing.

The blind and guided search strategies differ characteristically in how the search efficiency *Q* = *Q*(*v*_*tar*_, *ε*_*tar*_) depends on the migration parameters of the targets: While blind search (BLS, RMS) works better with fast and persistent targets, the opposite is true for guided search (TGS, SGS, CGS). In guided search, due to the optimization against a mixed set of targets, the search efficiency *Q* = *Q*(*v*_*tar*_, *ε*_*tar*_) remains approximately constant for most combinations of *v*_*tar*_ and *ε*_*tar*_. Only for targets that are simultaneously extremely fast and persistent does *Q* decline significantly. Assuming an experimental possibility to vary the migration properties of the targets, without affecting the immune cell or the properties of the chemo-attractant, this predicted difference in *Q* = *Q*(*v*_*tar*_, *ε*_*tar*_) offers an indirect possibility to distinguish between blind and guided search strategies.

Finally, our work suggests how to detect different search strategies of an immune cell by looking for characteristic patterns in the cell’s trajectory: In the case of temporal sensing, the immune cell will show alternating phases of low and high directional persistence, and the probability of the high persistence mode will increase whenever the immune cell approaches one of the targets. In the case of spatial sensing, the left- and right-turns of the immune cell will occur in such a way that they tend to align the cell into the direction of the closest target. Indeed, trajectories simulated with the models discussed in this work have already been used to validate different algorithms that can detect the presence of remote cell-cell interactions^45,46^.

In future work, our investigation could be improved and extended in various ways. For example, we have so far assumed that the immune cell is able to detect arbitrarily small concentrations (or differences between two concentrations) of the chemo-attractant. A lower detection limit may very well change the optimal search parameters and, accordingly, the associated search strategies. It would also be possible to go beyond the fast diffusion limit. The problem then becomes computationally more demanding, as it requires to solve the partial differential equation of the spreading and decaying chemo-attractant along with the motion of the cells. However, we have already demonstrated the feasibility of this approach in 2D (Supplemental Information [B]).

## Supplementary information

Supplementary Information

## Data Availability

All simulation programs and results are available online at http://tiny.cc/chemotacticpursuit.
