# Supplementary material for: On the efficiency of chemotactic pursuit - Comparing blind search with temporal and spatial gradient sensing
Source: Sci Rep. 2019 Oct 1;9:14091. doi: 10.1038/s41598-019-50514-4 (PMC6773759; doi:10.1038/s41598-019-50514-4)
Supplement: Supplementary file 1 — Supplementary Information [file 41598_2019_50514_MOESM1_ESM.pdf]

SUPPLEMENTAL INFORMATION

On the efficiency of chemotactic pursuit - Comparing blind search with temporal and spatial gradient sensing

Claus Metzner*

Department of Physics, Biophysics Group,

Friedrich-Alexander University Erlangen, Germany

(Dated: August 9, 2019)

* claus.metzner@gmail.com

[A] SIMULATION ALGORITHM

'Main simulation loop':

For all time steps n:

'Move agents' (see below).
'Remove killed targets' (see below).
'Update density field', according to Eq.(14).
'Adapt immune cell' (see below).

'Move agents':

For all cells c >=0 (including the immune cell c=0):

Draw step width $w_{c,n}$ from Rayleigh distribution, according to momentary speed parameter $v_{c,n}$ of cell c.

Draw turning angle $\Delta\Phi_{c,n}$ from uniform distribution, according to momentary persistence parameter $\varepsilon_{c,n}$ of cell c.

Compute magnitude $|\Delta\Phi_{c,n}|$ of turning angle.

Draw sign $s_{c,n}$ of turning angle from Bernoulli distribution, according to momentary 'right turn probability' $q_{R;c,n}$ of cell c (Note: $q_{R;c,n}$ = ½ for all target cells c>0).

Compute new cell position $\underline{r}_{c,n}$, according to Eq.(2).

For all cells c >= 0 (including the immune cell c=0) :

Apply periodic boundary conditions.

FIG. 1. *Description of the simulation algorithm: Main loop and the subroutine 'Move agents', which generates the blind random walk of the target cells and the (possibly goal directed) random walk of the immune cell.*

'Remove killed targets':

For all target cells c>0:

Compute distance d_{c0} to immune cell 0,
taking into account periodic boundary conditions.

If $d_{c0} > (r_{imm} + r_{tar})$: add target cell c to list of survivors.

Cell list := list of survivors.

'Adapt immune cell': (immune cell has number c=0)

Update previous value ρ^C_{n-1} at the central chemoattractant sensor.
Update present value ρ^C_n at the central chemoattractant sensor.
Compute temporal gradient $\Delta\rho^C_n$, according to Eq.(15).
Compute momentary 'approach mode probability' $q_{A;0,n}$,
according to Eq.(18).

Do with probability $q_{A;0,n}$:
Toggle immune cell speed $v_{0,n}$ between v_N and v_A.
Toggle immune cell persistence $\varepsilon_{0,n}$ between ε_N and ε_A.

Update value ρ^R_n at the right chemoattractant sensor.
Update value ρ^L_n at the left chemoattractant sensor.
Compute spatial gradient $\Delta\rho^{LR}_n$, according to Eq.(17).
Compute momentary 'right turn probability' $q_{R;0,n}$,
according to Eq.(20).

FIG. 2. *Description of the simulation algorithm: Subroutine 'Remove killed targets' deletes all target cells that had direct contact with the immune cell. Subroutine 'Adapt immune cell' updates the migration mode of the immune cell and its probability for right turns, depending on the density gradients of the chemoattractant.*

[B] BEYOND THE FAST DIFFUSION LIMIT

In this paper, we focused on the special case of a chemoattractant that is diffusing fast (large D) and decaying quickly (large k), so that the critical velocity $v_{crit} = \sqrt{kD}$ becomes much larger than the typical migration speed of the target cells. In this so-called 'fast diffusion limit', the global density profile $F_{2D}(\vec{r}, t)$ could be computed by the linear superposition of fixed 'kernels' $f_{2D}(\vec{r})$, one centered around each of the target cells.

In the general case, it is necessary to simulate the temporal evolution of the density field, which is governed by Eq. (6), along with the motion of the immune and target cells. The main structure of the simulation algorithm (Supplemental Information [A]) remains the same, but the subroutine 'Update density field' becomes more elaborate:

The most simple way to solve the partial differential equation Eq. (6) is based on a discretization of the simulation area into a regular grid of quadratic patches with linear patch size ΔL, so that the continuous density field $F_{2D}(\vec{r}, t)$ is represented by the discrete array $F_{2D}(X, Y, n)$. In every simulation time step n, the chemoattractant density in each of the patches is changing due to the generation term, the diffusion term, and the decay term in Eq. (**??**). The generation term effectively adds, in every time step, a certain amount $\hat{g}$ of density to all patches (X, Y) in which a target cell is presently located. The diffusion term removes an amount $\hat{D}\ F_{2D}(X, Y, n)$ from every patch (X, Y), and redistributes this amount in equal parts to the four nearest neighbor patches $(X+1, Y)$, $(X, Y+1)$, $(X-1, Y)$ and $(X, Y-1)$. Finally, the decay term multiplies the density at every patch (X, Y) with a certain factor $\hat{m}$ that is smaller than one. Given the spatial discretization length ΔL and the temporal discretization time Δt_{sim}, the three effective simulation parameters $\hat{g}$, $\hat{D}$ and $\hat{m}$ can be computed from the actual model parameters g, D and k. However, we will use the effective parameters in the following two examples.

In the examples, we consider target cells that move diffusively ($\epsilon_{tar} = 0$) and at low speed ($v_{tar} = 1$). The immune cell moves with the same speed ($v_{imm} = 1$), but uses temporal gradient sensing to switch its directional persistence between $\epsilon_{imm} = 0.7$ in the approach mode and $\epsilon_{imm} = 0$ in the normal migration mode. Response coefficients were $c_{A0} = 10$ and $c_{A1} = 500$. The effective parameter for chemoattractant generation was $\hat{g} = 1$, and the effective diffusion was ($\hat{D} = 0.02$).

If the chemoattractant decays relatively quickly ($\hat{m} = 0.9$, Fig. 3), the situation is similar

to the fast diffusion limit: Each target cell is surrounded by a distinct 'cloud', and the gradient within these clouds guides the immune cell reliably to the targets themselves.

A qualitatively different situation arises if the chemoattractant decays relatively slowly ($\hat{m} = 0.999$, Fig. 4). In this case, the emissions of several targets 'bleed together' and form flat density plateaus. It is difficult for the immune cells to pick up density gradients within these plateaus.

These example demonstrate that the efficiency Q of different search strategies (such as TGS and SGS) will probably depend in a complex way on the diffusion constant D and decay rate k of the chemoattractant. While it would be interesting to map out the complete 'phase diagram' $Q = Q(v_{tar}, \epsilon_{tar}, D, K)$, this is clearly beyond the scope of this paper.

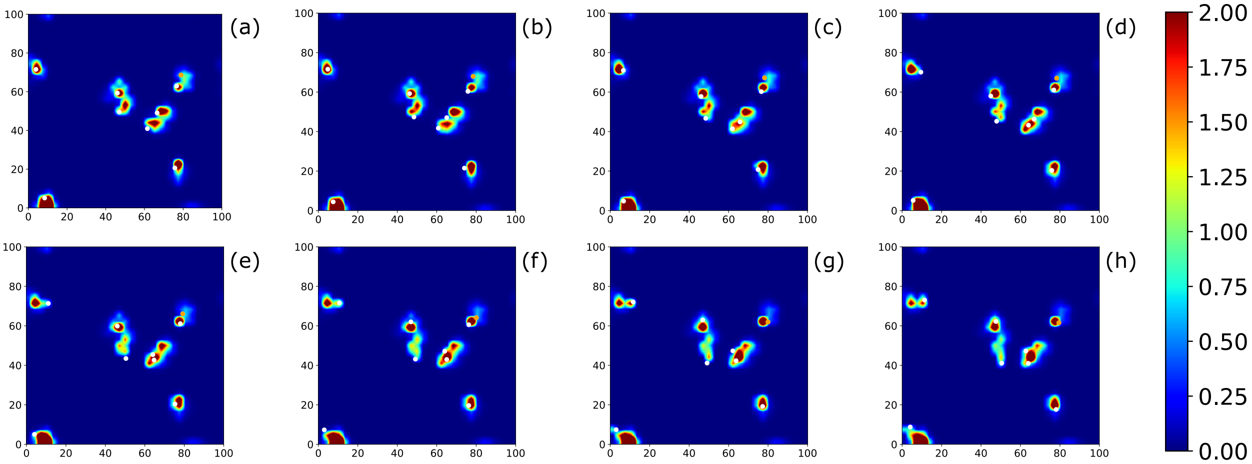

FIG. 3. *System evolution in a case of slow diffusion ($\hat{D} = 0.02$) and fast decay ($\hat{m} = 0.9$) of the chemoattractant. The density of chemoattractant is color-coded, white dots represent target cells, the orange dot is the immune cell. Panels (a)-(h) correspond to time steps 120-127 of the simulation. Due to the fast decay of the chemoattractant, each target cell is surrounded by a distinct 'cloud', and the gradient within the clouds guides the immune cell to its targets.*

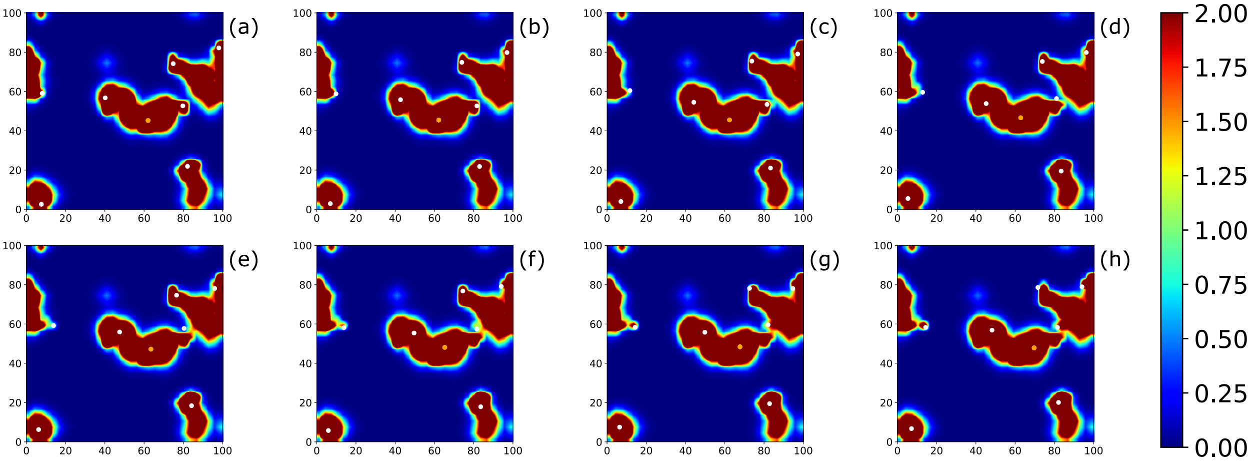

FIG. 4. *System evolution in a case of slow diffusion ($\hat{D} = 0.02$) and slow ($\hat{m} = 0.999$) decay of the chemoattractant. The density of chemoattractant is color-coded, white dots represent target cells, the orange dot is the immune cell. Panels (a)-(h) correspond to time steps 120-127 of the simulation. Due to the slow decay of the chemoattractant, the emissions of several targets 'bleed together' and form flat density plateaus. It is difficult for the immune cells to pick up density gradients within these plateaus.*
